# Effects of the COVID-19 pandemic and ‘Find Cancer Early’ campaign on cancer symptom knowledge in regional Western Australia

**DOI:** 10.1093/pubmed/fdaf083

**Published:** 2025-07-13

**Authors:** Ying Ru Feng, Derrick Lopez, Sarah V Ward, Cassandra Clayforth, Clover Maitland, Hussam Al-Hakimi, Melissa Ledger, Elizabeth Sorial, David B Preen

**Affiliations:** School of Population and Global Health, The University of Western Australia, Stirling Hwy, Crawley, WA 6009, Australia; School of Population and Global Health, The University of Western Australia, Stirling Hwy, Crawley, WA 6009, Australia; School of Population and Global Health, The University of Western Australia, Stirling Hwy, Crawley, WA 6009, Australia; UWA Medical School, The University of Western Australia, Stirling Hwy, Crawley, WA 6009, Australia; Cancer Council Western Australia, Bagot Road, Subiaco, WA 6008, Australia; Centre for Behavioural Research in Cancer, Cancer Council Victoria, Victoria Pde, East Melbourne, VIC 3002, Australia; School of Human Sciences, The University of Western Australia, Stirling Hwy, Crawley, WA 6009, Australia; Cancer Council Western Australia, Bagot Road, Subiaco, WA 6008, Australia; Cancer Council Western Australia, Bagot Road, Subiaco, WA 6008, Australia; School of Population and Global Health, The University of Western Australia, Stirling Hwy, Crawley, WA 6009, Australia; School of Population and Global Health, The University of Western Australia, Stirling Hwy, Crawley, WA 6009, Australia

**Keywords:** cancer awareness, cancer symptoms, COVID-19, health campaign, regional health

## Abstract

**Background:**

Awareness of cancer and its symptoms may have declined in 2020 following the onset of the COVID-19 pandemic. We examined the effect of the COVID-19 pandemic and awareness of the ‘Find Cancer Early’ mass media campaign on the knowledge of common cancer symptoms in regional Western Australian (WA) residents aged 40 years and over.

**Methods:**

Campaign materials from ‘Find Cancer Early’ included the ‘Rural Doctors’ video advertisement and the ‘Yellow Checklist’. Multivariable log-binomial regression analyses were undertaken using data from the annual post-campaign evaluations surveys between 2018 and 2020, which included information on campaign awareness, cancer symptom knowledge, and sociodemographic factors.

**Results:**

The number of cancer symptoms recalled (mean (M): 1.32 vs 1.72; *P* < .001) and the proportion of participants with better cancer symptom knowledge (16.9% vs 25.7%; *P* < .001) were lower in 2020 than the pre-pandemic time-period. Campaign awareness (prevalence ratio (PR) = 1.99; *P* < .001) and females (PR = 1.28; *P* = .002) were associated with better cancer symptom knowledge, while the pandemic time-period (PR = 0.59; *P* < .001), older age (PR = 0.57; *P* < .001), and a previous cancer diagnosis (PR = 0.76; *P* = .007) were associated with poorer knowledge.

**Conclusions:**

Despite the ongoing pandemic, campaign awareness was associated with better knowledge of common cancer symptoms in regional WA. Regional subpopulations, including males and older adults, should be targeted for future campaigns.

## Introduction

Health inequities between regional and metropolitan areas are still evident in cancer outcomes in Australia.^1^^,^^2^ It was previously reported that survival for all cancers decreased with increasing residential remoteness, with major cities having a 63% 5-year survival, compared with 55% in very remote areas.^2^^,^^3^ Poorer cancer outcomes for regional residents can be attributed to several factors, including disparities in symptom appraisal, delays in help-seeking and diagnosis, and barriers to accessing care and treatment.^4–7^

The ‘Find Cancer Early’ campaign (hereafter referred to as ‘the Campaign’), developed by the Cancer Council Western Australia, is an annual and ongoing mass media campaign on cancer symptom awareness and early diagnosis.^8^ It is targeted towards regional Western Australian residents aged 40 years and above, aiming to inform about the symptoms of common cancers and reduce barriers to seeking help from doctors regarding these symptoms. Exploratory research with recently diagnosed rural cancer patients guided the Campaign and found that core characteristics of the rural Australian population, including optimism, stoicism, and machismo, in combination with reduced access to healthcare, contributed to delayed cancer presentations.^5^ An early evaluation of the Campaign (from November 2011 to July 2013) reported that it was successful in raising awareness about cancer signs and symptoms in the target audience.^9^

While cancer remains the leading health burden in Australia,^10^ the COVID-19 pandemic emerging in 2020 became a competing health priority for many chronic conditions.^11^ Restrictions enforced during the pandemic included travel restrictions, social distancing, and capacity limits, which likely contributed to changes in health service use. Nationally, there were 178 000 fewer cancer-related diagnostic and treatment service claims in 2020, which was 8% lower than the estimated projected number based on trends from 2017 to 2019.^12^ Given the global attention dedicated towards the COVID-19 pandemic, health promotion campaigns for other conditions may have been overshadowed during this period. The primary aim of this study was to examine the effect of the COVID-19 pandemic and awareness of the ‘Find Cancer Early’ campaign on the knowledge of common cancer symptoms in regional Western Australian residents aged 40 years and over. A secondary aim was to investigate the association between sociodemographic factors with cancer symptom knowledge.

## Methods

### Study design

This study was conducted in the regional and remote areas of Western Australia (WA), determined by the WA Country Health Service: (1) South West; (2) Wheatbelt; (3) Great Southern; (4) Goldfields-Esperance; (5) Mid West-Gascoyne; (6) Pilbara; and (7) Kimberley. WA is the largest state by area and the most geographically vast. In 2020, WA had a population of 2.66 million, with 567 951 people (21.3%) residing in regional or remote areas.^13^

Data from the annual ‘Find Cancer Early’ post-campaign evaluation surveys between 2018 and 2020 were used in this study. The 2018 and 2019 surveys were combined and categorised as ‘pre-pandemic’ waves. The 2020 survey formed the ‘pandemic’ wave, as it occurred after the onset of the COVID-19 pandemic.

### Recruitment and participants

Regional WA households were identified from the Electronic White Pages (in 2018 and 2019) and Australian Residential Database (in 2020) and a participant from each household was contacted for recruitment by the Survey Research Centre at Edith Cowan University. For each annual survey, researchers aimed to recruit the following participant quotas: (1) 67% aged 40–64 years and 33% aged 65+ years; (2) 50% males and 50% females; and (3) 14% from each geographic region.

Survey data were collected from consenting participants using computer-assisted telephone interviewing. These surveys collected information on sociodemographic factors (including age, gender, region, and cancer diagnosis history), knowledge of common cancer symptoms, awareness and opinions regarding the Campaign, and perspectives on earlier detection of cancer symptoms (using a 4-point Likert scale). The survey also contained adapted questions previously used to evaluate the public awareness of other Cancer Council campaigns.

### ‘Find Cancer Early’ campaign and awareness

Between 2018 and 2020, Cancer Council Western Australia implemented the following campaign materials as part of the ‘Find Cancer Early’ campaign: (1) the ‘Rural Doctors’ video advertisement, aired on television and digital/online platforms throughout regional WA; (2) four radio advertisements aired on free-to-air radio stations throughout regional WA; and (3) the ‘Yellow Checklist’ printed in local rural newspapers, as well as posters distributed by Cancer Council Western Australia Regional Education Officers. Supplementary Table S1 summarises these campaign materials. Regional WA residents previously provided feedback on campaign materials as part of the Campaign’s development.

In the survey, participants were asked to recall and describe any health advertisements about cancer that they had seen or heard in the last year. Those who correctly described the ‘Rural Doctors’ advertisement were categorized as having unprompted awareness of the ‘Rural Doctors’ advertisement. Participants who reported not having seen or heard any advertisements or had not correctly described the ‘Rural Doctors’ advertisement were then provided a description of this advertisement and asked whether they recalled seeing it. Those who responded that they had seen it were categorised as having prompted awareness of the ‘Rural Doctors’ advertisement. All participants were then asked if they had seen a health advertisement which included a yellow checklist of cancer symptoms. Those who confirmed this were categorised as having awareness of the ‘Yellow Checklist’. Participants were not specifically asked about radio advertisements.

Participants who had awareness of either the ‘Rural Doctors’ advertisement or the ‘Yellow Checklist’ were then asked if they agreed (responded with ‘strongly agreed’ or ‘agreed’) or disagreed (responded with ‘strongly disagreed’ or ‘disagreed’) that the Campaign was (1) easy to understand, (2) taught them something new, (3) made them feel uncomfortable, (4) believable, (5) relevant, and (6) offensive.

### Knowledge of common cancer symptoms

Knowledge of common cancer symptoms was based on participants’ ability to recall the 10 most common predictive symptoms of the 5 most common cancers (prostate, breast, skin, bowel, and lung) promoted throughout the Campaign. These symptoms included (1) coughing up blood; (2) nagging cough; (3) becoming more short of breath; (4) blood in pee; (5) blood in poo; (6) problems peeing; (7) runny or looser poo (diarrhoea); (8) an unusual pain, lump, or swelling anywhere in your body; (9) unexplained weight loss; and (10) a new or changed spot on your skin. Participants were asked, *‘Can you please tell me what you think are the most common (signs and) symptoms of cancer?’* before being asked the questions on campaign awareness. Those who recalled three or more symptoms were categorised as having *‘better cancer symptom knowledge’,* and those unable to recall at least three symptoms were categorised as having *‘poorer cancer symptom knowledge’*.

### Statistical analyses

Descriptive statistics summarised the sociodemographic factors, campaign awareness and opinions, perspectives on earlier detection of cancer symptoms, and cancer symptom knowledge. Chi-squared tests and Student’s *t*-tests were used to test for differences between the pre-pandemic and pandemic waves.

Multivariable log-binomial regression models examined the association between the pandemic, campaign awareness, and sociodemographic factors with the outcome of cancer symptom knowledge. The pandemic was included as a binary pre-pandemic/pandemic time-period variable in all regression models. Separate regression models were undertaken which included different aspects of campaign awareness. The overall model examined the relationship between awareness of either the ‘Rural Doctors’ advertisement or the ‘Yellow Checklist’ with cancer symptom knowledge, compared with no awareness of either of these campaign materials. Other regression models examined the association of awareness of both the ‘Rural Doctors’ advertisement and the ‘Yellow Checklist’ (Model A); awareness of only the ‘Rural Doctors’ advertisement (Model B); and awareness of only the ‘Yellow Checklist’ (Model C), with cancer symptom knowledge. The relationship between unprompted awareness of the ‘Rural Doctors’ advertisement and cancer symptom knowledge, compared with prompted awareness, was also examined (Model D). Regression models initially adjusted for age (40–64 years/65+ years), gender (male/female), geographical region, cancer diagnosis history (previous cancer diagnosis/none), and the interaction term between pandemic time-period and campaign awareness. However, Type III Tests showed that neither region nor the interaction term had a significant overall effect and were subsequently removed from the final models. All statistical analyses were undertaken using SAS v9.4. The level of statistical significance was set at *P* <.05.

### Ethics

Ethics approval was obtained from the Human Ethics Committee at The University of Western Australia (2022/ET000404).

## Results

### Study population characteristics

The study population consisted of 3810 participants, with 2758 (72.4%) and 1052 (27.6%) from the pre-pandemic and pandemic waves, respectively. Sociodemographic factors and perspectives on earlier detection of cancer symptoms were similar between the waves and are summarised in Table 1 (and Supplementary Table S2). The majority were aged 40–64 years (65.6%), female (53.1%), and had no previous cancer diagnosis (77.3%). Of those with a previous cancer diagnosis, skin cancer was the most frequently diagnosed cancer (45.8%).

**Table 1:** Sociodemographic factors, cancer diagnosis history, and perspectives on earlier detection of cancer symptoms.

	All waves	Pre-pandemic waves	Pandemic wave	*P* value^a^
	*n* = 3810	*n* = 2758	*n* = 1052	
	*n* (%)	*n* (%)	*n* (%)	
**Age group**				
40–64 years	2498 (65.6)	1830 (66.4)	668 (63.5)	.097
65+ years	1312 (34.4)	928 (33.6)	384 (36.5)	
**Gender**				
Male	1788 (46.9)	1283 (46.5)	505 (48.0)	.412
Female	2022 (53.1)	1475 (53.5)	547 (52.0)	
**Region**				
Wheatbelt	542 (14.2)	391 (14.2)	151 (14.4)	1.000
Great Southern	540 (14.2)	390 (14.1)	150 (14.3)	
Goldfields-Esperance	541 (14.2)	391 (14.2)	150 (14.3)	
Mid West-Gascoyne	542 (14.2)	392 (14.2)	150 (14.3)	
South West	556 (14.6)	405 (14.7)	151 (14.4)	
Kimberley	540 (14.2)	390 (14.1)	150 (14.3)	
Pilbara	549 (14.4)	399 (14.5)	150 (14.3)	
**Previous cancer diagnosis**				
No	2946 (77.3)	2150 (78.0)	796 (75.7)	.131
Yes	864 (22.7)	608 (22.0)	256 (24.3)	
**Type of cancer** ^**b**^				
Prostate	92 (10.8)	60 (10.0)	32 (12.5)	.251
Breast	126 (14.8)	85 (14.1)	41 (16.2)	.438
Skin	391 (45.8)	271 (45.1)	120 (47.4)	.531
Bowel	63 (7.4)	47 (7.8)	16 (6.3)	.445
Lung	18 (2.1)	14 (2.3)	4 (1.6)	.487
Other	193 (22.6)	143 (23.8)	50 (19.7)	.198
**Detecting cancer at an early stage means it can be treated more successfully**				
Agree	3677 (98.3)	2662 (98.3)	1015 (98.1)	.572
Disagree	65 (1.7)	45 (1.7)	20 (1.9)	
**Campaigns that raise awareness of cancer symptoms lead to earlier detection of cancer**				
Agree	2701 (96.9)	1935 (96.8)	766 (97.1)	.743
Disagree	86 (3.1)	63 (3.2)	23 (2.9)	
**More should be done in your region to raise awareness of the symptoms of cancer**				
Agree	1892 (69.2)	1332 (67.9)	560 (72.5)	.018^*^^*^
Disagree	842 (30.8)	630 (32.1)	212 (27.5)	
**The state government should continue to fund cancer symptom awareness campaigns targeted towards regional people** ^**c**^				
Agree		–	727 (89.3)	–
Neutral	–	–	63 (7.7)	
Disagree	–	–	24 (3.0)	

^**^ *P* < .05

a Chi-squared tests were used to determine for differences between the pre-pandemic and pandemic waves.

b Question restricted to participants who had reported having previously been diagnosed with cancer.

c Question only included in 2020 survey.

Dashes mean not applicable

### Perspectives on earlier detection of cancer symptoms

Overall, 98.3% and 96.9% of participants agreed that detecting cancer at an early stage meant that it could be treated more successfully, and that raising awareness of cancer symptoms lead to earlier detection of cancers, respectively. While most agreed that *‘more should be done in regional areas to raise awareness of the symptoms of cancer’*, the proportion agreeing was significantly higher in the pandemic wave (72.5%) than the pre-pandemic waves (67.9%; *P* = .018). Additionally, the majority from the pandemic wave (89.3%) supported continued state government funding for a cancer symptom awareness campaign to address the worse cancer outcomes that regional people experience.

### Awareness and opinions on the ‘Find Cancer Early’ campaign

Awareness of the ‘Rural Doctors’ advertisement and the ‘Yellow Checklist’ and opinions about the Campaign are summarised in Table 2 (and Supplementary Tables S3 and S4). More participants had seen or heard an advertisement about cancer (not specifically from the ‘Find Cancer Early’ campaign) from the pandemic wave (74.8%) than the pre-pandemic waves (71.1%; *P* = .021). However, awareness of ‘Find Cancer Early’ campaign was similar between the pre-pandemic (73.2%) and pandemic waves (75.6%; *P* = .132). While those from the pandemic wave also had higher awareness (73.6% vs 70.9%; *P* = .100) and unprompted awareness (28.9% vs 26.1%; *P* = .073) of the ‘Rural Doctors’ advertisement than the pre-pandemic waves, these did not reach statistical significance. Awareness of the ‘Yellow Checklist’ was also statistically similar between the waves (12.0%; *P* = .984).

**Table 2:** Awareness of the ‘Find Cancer Early’ campaign.

	All waves	Pre-pandemic waves	Pandemic wave	*P* value^a^
	*n* = 3810	*n* = 2758	*n* = 1052	
	*n* (%)	*n* (%)	*n* (%)	
**Seen or heard an advertisement about cancer** ^**b**^	2747 (72.1)	1960 (71.1)	787 (74.8)	.021^*^^*^
**Awareness of campaign material** ^**c**^	2813 (73.8)	2018 (73.2)	795 (75.6)	.132
**No awareness of campaign material** ^**c**^	997 (26.2)	740 (26.8)	257 (24.4)	.132
**‘Rural Doctors’ advertisement**				
Unprompted awareness	1023 (26.9)	719 (26.1)	304 (28.9)	.073
Prompted awareness	1706 (44.8)	1236 (44.8)	470 (44.7)	.939
Awareness (unprompted or prompted awareness)	2729 (71.6)	1955 (70.9)	774 (73.6)	.100
**‘Yellow Checklist’**				
Awareness	457 (12.0)	331 (12.0)	126 (12.0)	.984
**Opinions about the ‘Find Cancer Early’ campaign** ^**d**^				
Easy to understand	2757 (99.3)	1990 (99.4)	767 (99.0)	.226
Taught something new	1168 (42.4)	831 (42.0)	337 (43.5)	.454
Uncomfortable (to view/watch)	245 (8.8)	174 (8.6)	71 (9.1)	.719
Believable	2748 (98.7)	1983 (98.8)	765 (98.5)	.536
Relevant	2302 (83.0)	1668 (83.4)	634 (81.8)	.317
Offensive	63 (2.3)	43 (2.1)	20 (2.6)	.476

^**^ *P* < .05

a Chi-squared tests were used to determine for differences between the pre-pandemic and pandemic waves.

b Advertisement not necessarily specific to the ‘Find Cancer Early’ campaign.

c Either the ‘Rural Doctors’ advertisement or the ‘Yellow Checklist’.

d Questions restricted to participants who had awareness of the ‘Find Cancer Early’ campaign.

Campaign opinions were also not significantly different between the two waves. Overall, the majority felt it was easy to understand (99.3%), believable (98.7%), relevant (83.0%), was not uncomfortable to view (91.2%), or offensive (97.7%), but that it had not taught them something new (57.6%).

### Knowledge of common cancer symptoms

Participants’ knowledge of common cancer symptoms is summarised in Table 3 (and Supplementary Table S5). Participants from the pandemic wave recalled fewer symptoms (mean (M) = 1.3; standard deviation (SD) = 1.2) than those from the pre-pandemic waves (M = 1.7; SD = 1.4; *P* < .001) and were more likely to have poorer cancer symptom knowledge (83.1% vs 74.3%; *P* < .001). Specifically, fewer participants were able to recall the following symptoms in the pandemic wave, compared with the pre-pandemic waves: nagging cough (4.0% vs 6.9%; *P* < .001); blood in pee (8.7% vs 15.1%; *P* < .001); blood in poo (17.1% vs 23.4%; *P* < .001); an unusual pain, lump, or swelling anywhere in your body (46.5% vs 53.3%; *P* < .001); and a new or changed spot on your skin (24.2% vs 38.2%; *P* < .001).

**Table 3:** Knowledge on the common symptoms of cancer.

	All waves	Pre-pandemic waves	Pandemic wave	*P* value^a^
	*n* = 3804	*n* = 2756	*n* = 1048	
	*n* (%) or M ± SD	*n* (%) or M ± SD	*n* (%) or M ± SD	
**Number of cancer symptoms recalled**	1.6 ± 1.3	1.7 ± 1.4	1.3 ± 1.2	<.001^*^^*^
**Cancer symptom knowledge**				
Poorer (recalled <3 symptoms)	2919 (76.7)	2048 (74.3)	871 (83.1)	<.001^*^^*^
Better (recalled 3+ symptoms)	885 (23.3)	708 (25.7)	177 (16.9)	
**Cancer symptom recalled**				
Coughing up blood	145 (3.8)	110 (4.0)	35 (3.3)	.348
Nagging cough	232 (6.1)	190 (6.9)	42 (4.0)	<.001^*^^*^
Becoming more short of breath	125 (3.3)	89 (3.2)	36 (3.4)	.750
Blood in pee	506 (13.3)	415 (15.1)	91 (8.7)	<.001^*^^*^
Blood in poo	825 (21.7)	646 (23.4)	179 (17.1)	<.001^*^^*^
Problems peeing	77 (2.0)	61 (2.2)	16 (1.5)	.179
Runny/looser poo (diarrhoea)	94 (2.5)	67 (2.4)	27 (2.6)	.797
An unusual pain, lump, or swelling anywhere in your body	1957 (51.4)	1470 (53.3)	487 (46.5)	<.001^*^^*^
Unexplained weight loss	868 (22.8)	648 (23.5)	220 (21.0)	.098
A new or changed spot on your skin	1306 (34.3)	1052 (38.2)	254 (24.2)	<.001^*^^*^

Abbreviations: M, mean; SD, standard deviation.

^*^^*^ *P* < .05.

a Independent *t*-tests were used to determine for differences between the pre-pandemic and pandemic waves for the number of cancer symptoms recalled; chi-squared tests and Fisher’s exact tests were used to determine for differences between the pre-pandemic and pandemic waves for cancer symptom knowledge and cancer symptom recalled.

Table 4 summarises the multivariable log-binomial regression models on cancer symptom knowledge. The overall model showed that awareness of either the ‘Rural Doctors’ advertisement or the ‘Yellow Checklist’ was associated a two-fold higher likelihood (prevalence ratio (PR) = 1.99; 95% confidence intervals (CI): 1.64, 2.42; *P* < .001) of having better cancer symptom knowledge compared with no awareness. Additionally, the pandemic time-period (PR = 0.58; 95% CI: 0.48, 0.70; *P* < .001), older age (PR = 0.57; 95% CI: 0.48, 0.68; *P* < .001), and a previous cancer diagnosis (PR = 0.76; 95% CI: 0.63, 0.93; *P* = 0.007) were associated with poorer cancer symptom knowledge. However, females were 1.3 times more likely (PR = 1.28; 95% CI: 1.09, 1.49; *P* = .002) to have better cancer symptom knowledge than males.

**Table 4:** Prevalence ratio for the outcome of knowledge on the common symptoms of cancer.

	Overall model		Model A		Model B		Model C		Model D	
	PR (95% CI)	*P* value	PR (95% CI)	*P* value	PR (95% CI)	*P* value	PR (95% CI)	*P* value	PR (95% CI)	*P* value
**Awareness of either campaign materials** (ref: No awareness of either advertisement)	1.99 (1.64, 2.42)	<.001^*^^*^	**–**	**–**	**–**	**–**	**–**	**–**	**–**	**–**
**Awareness of both campaign materials** (ref: No awareness of either advertisement)	–	–	2.38 (1.78, 3.18)	<.001^*^^*^	**–**	**–**	**–**	**–**	**–**	**–**
**Awareness of the ‘Rural Doctors’ advertisement** (ref: No awareness of either advertisement)	–	–	**–**	**–**	1.96 (1.60, 2.39)	<.001^*^^*^	–	–	**–**	**–**
**Awareness of the ‘Yellow Checklist’ only** (ref: No awareness of either advertisement)	–	–	–	–	**–**	**–**	0.79 (0.27, 2.30)	.662	–	–
**Unprompted awareness of ‘Rural Doctors’ advertisement** (ref: Prompted awareness of ‘Rural Doctors’ advertisement)	–	–	**–**	**–**	**–**	**–**	–	–	2.68 (2.24, 3.20)	<.001^*^^*^
**Pandemic** (ref: Pre-pandemic)	0.58 (0.48, 0.70)	<.001^*^^*^	0.79 (0.57, 1.09)	.155	0.55 (0.45, 0.68)	<.001^*^^*^	0.34 (0.12, 0.98)	.046^*^^*^	0.53 (0.43, 0.66)	<.001^*^^*^
**65+ years** (ref: 40–64 years)	0.57 (0.48, 0.68)	<.001^*^^*^	0.54 (0.39, 0.74)	<.001^*^^*^	0.58 (0.48, 0.70)	<.001^*^^*^	0.58 (0.40, 0.86)	.007^*^^*^	0.62 (0.51, 0.76)	<.001^*^^*^
**Female** (ref: Male)	1.28 (1.09, 1.49)	.002^*^^*^	1.37 (1.04, 1.81)	.026^*^^*^	1.25 (1.06, 1.48)	.009^*^^*^	1.12 (0.80, 1.58)	.498	1.26 (1.06, 1.51)	.011^*^^*^
**Previous cancer diagnosis** (ref: No previous diagnosis)	0.76 (0.63, 0.93)	.007^*^^*^	0.79 (0.56, 1.13)	.195	0.74 (0.60, 0.92)	.007^*^^*^	0.77 (0.49, 1.22)	.273	0.79 (0.63, 0.98)	.036^*^^*^

^*^^*^ *P* < .05.

Abbreviations: PR, prevalence ratio; CI, confidence interval; ref, reference group.

Overall Model: includes awareness of either the ‘Rural Doctors’ advertisement or the ‘Yellow Checklist’; Model A: includes awareness of both the ‘Rural Doctors’ advertisement and the ‘Yellow Checklist’; Model B: includes awareness of the ‘Rural Doctors’ advertisement only; Model C: includes awareness of the ‘Yellow Checklist’ only; Model D: includes unprompted awareness of the ‘Rural Doctors’ advertisement.

Dashes mean not applicable

Awareness of both the ‘Rural Doctors’ advertisement and the ‘Yellow Checklist’, and awareness of only the ‘Rural Doctors’ advertisement were associated with 2.4-times (Model A: PR = 2.38; 95% CI: 1.78, 3.18; *P* < .001) and 2.0-times (Model B: PR = 1.96; 95% CI: 1.60, 2.39; *P* < .001) greater likelihood of better knowledge of cancer symptoms, compared with no awareness of campaign materials. In contrast, awareness of only the ‘Yellow Checklist’ was not associated with cancer symptom knowledge (Model C: *P* = .662). Unprompted awareness of the ‘Rural Doctors’ advertisement was associated with 2.7-times (Model D: PR = 2.68; 95% CI: 2.24, 3.20; *P* < .001) greater likelihood of better cancer symptom knowledge compared with only prompted awareness.

## Discussion

### Main findings of this study

The current study examined the effect of the COVID-19 pandemic and awareness of the ‘Find Cancer Early’ campaign on the knowledge of common cancer symptoms in regional Western Australia. While campaign awareness throughout the study period consistently remained >70%, a decline in knowledge of common cancer symptoms was observed following the onset of the pandemic in 2020. The average number of symptoms recalled was lower and fewer participants were reported to have better cancer symptom knowledge in the pandemic wave than the pre-pandemic waves. However, in addition to the pre-pandemic time-period, having awareness about the Campaign was still associated with better cancer symptom knowledge, while sociodemographic factors, including males, older adults, and a previous cancer diagnosis, were identified to be associated with poorer knowledge.

### What is already known on this topic

The COVID-19 pandemic had a significant impact on the health system. Acute and urgent health services were often prioritised due to the pandemic, disrupting services related to chronic disease management and prevention.^14^ The intense global focus on COVID-19 likely overshadowed competing health concerns, including cancer, leading them to be perceived as a lower priority, which could explain the decline in cancer symptom knowledge.

The current study found associations between several sociodemographic factors and the knowledge of common cancer symptoms, including older age being linked to poorer symptom knowledge. A systematic review by Jones *et al*.^15^ supports this finding, as the authors previously identified several studies suggesting that older adults may be less aware of cancer symptoms. Given that increasing age is also a risk factor for comorbidities, new symptoms and bodily changes may also concerningly be attributed to pre-existing medical conditions, which could delay help-seeking behaviours and diagnosis.^15–17^ Alternatively, older adults and individuals with comorbidities could also experience the opposite due the surveillance effect, where cancers are diagnosed earlier due to more frequent health service contact. However, Gurney *et al*.^18^ found no evidence of this phenomenon, suggesting that comorbidities may rather distract patients, allowing early symptoms of cancers to go unnoticed.

Males were also more likely to have poorer cancer symptom knowledge than females, similar to findings from a previous study undertaken in an English population.^19^ Niksic *et al*.^19^ reported that women were more likely to recognise cancer symptoms compared with men, suggesting that this could be due to the more dominant role that females have in health and caregiving. Similarly, Fish *et al*.^20^ conducted semi-structured interviews with Australian men recently diagnosed with cancer and found that many participants believed that women had greater awareness about their health status and relied on their female partners regarding the detection of cancer symptoms and engaging in help-seeking.

Additionally, we observed that a previous cancer diagnosis was associated with poorer cancer symptom knowledge. Much less is known about this relationship in the literature. One explanation could be that cancer survivors may also experience cancer-related post-traumatic stress,^21^ leading them to distance themselves away from additional cancer information. However, further investigation regarding this relationship is warranted.

### What this study adds

The symptoms promoted in the ‘Find Cancer Early’ campaign are critical for early cancer detection, yet can easily be self-monitored frequently (e.g. when going to the toilet). Remaining vigilant about bodily changes may have been more crucial during 2020 as regular monitoring of symptoms through health professionals and screening services experienced disruptions due to the pandemic,^22^ particularly in regional and remote areas.^23^ The current study highlights a potential concern regarding the decline in cancer symptom knowledge and whether this trend continues over time beyond the initial onset of the pandemic. Given the effectiveness of self-monitoring for earlier cancer detection, long-term effects of the pandemic on cancer outcomes may remain unknown until much later.

Our findings also suggest that the ‘Find Cancer Early’ campaign can be an effective tool for informing regional residents about cancer symptoms. Awareness of the ‘Rural Doctors’ advertisement, particularly unprompted awareness, was consistently linked with better cancer symptom knowledge. The Campaign was also well received as being relevant and believable. While the minority reported that the Campaign had taught them something new, this was not unexpected as the Campaign has been delivered annually since 2014 in regional areas. It suggests that the Campaign serves to reinforce important health messages, in addition to being an educational tool. Despite the ongoing pandemic, the majority supported regional campaigns on raising awareness about cancer symptoms. Furthermore, the current study has also identified regional subpopulations, such as older adults, males, and those with a previous cancer diagnosis, as potential candidates for future targeted campaigns and intervention. Development of advertising materials with community members from these subpopulations could also increase the effectiveness of the campaign.

### Limitations of this study

While the current study used a large sample from various regional WA communities, a few limitations have been identified. First, we did not include a control group of regional residents residing in areas where the Campaign was not delivered. However, an earlier evaluation of the ‘Find Cancer Early’ campaign, which included a control group, found that symptom recall was higher in the campaign regions than control regions. Other sociodemographic information (e.g. employment, education) or information on health literacy were not collected in the surveys and therefore not adjusted for in the analyses. Additionally, participants were not specifically asked about awareness of radio advertisements, which discussed the symptoms of common cancers. Therefore, participants categorised as having no awareness of campaign materials may have been exposed to the Campaign through this platform, leading to more conservative estimates for cancer symptom knowledge. However, it is likely that those who heard radio advertisements would have also viewed the video/TV advertisement.

The main ‘Find Cancer Early’ TV/video advertisement changed from the ‘Rural Doctors’ advertisement (airing from 2014 to 2020, inclusive) to the ‘Regional Champions’ advertisement (airing from 2021 onwards) which featured personal testimonies. Therefore, campaign years which featured the ‘Rural Doctors’ advertisement were only included in this study to ensure that changes in outcomes were not due to changes in the advertisement, and it is unknown if findings may be generalisable to the other campaign years. As the Campaign runs over consecutive years, participants may have been exposed to and recalled campaign material from prior years.

## Conclusion

Although the decline in cancer symptom knowledge was evident during the pandemic, the ‘Find Cancer Early’ campaign was still successful at informing and educating regional residents about the common symptoms of cancers, which could assist in earlier cancer detection, more successful treatment, and improved survival. Regional subpopulations, including older adults, males, and those with a previous cancer diagnosis, should be considered for future campaigns which raise awareness about cancer symptoms.

## Supplementary Material

Supplementary_materials_fdaf083

## Data Availability

The data that support the findings of this study are available on request from the corresponding author. The data are not publicly available due to privacy or ethical restrictions.

## Notes

**Ying Ru Feng,** Research Fellow

**Derrick Lopez,** Research Fellow

**Sarah V. Ward,** Senior Research Fellow

**Cassandra Clayforth,** Regional Education Manager

**Clover Maitland,** Senior Research Fellow

**Hussam Al-Hakimi,** Find Cancer Early Senior Coordinator

**Melissa Ledger,** Cancer Prevention and Research Director

**Elizabeth Sorial,** PhD Student

**David B. Preen,** Professor and Chair in Public Health
