# Supplementary material for: Effects of the COVID-19 pandemic and ‘Find Cancer Early’ campaign on cancer symptom knowledge in regional Western Australia
Source: J Public Health (Oxf). 2025 Jul 13;47(4):737–46. doi: 10.1093/pubmed/fdaf083 (PMC12669984; doi:10.1093/pubmed/fdaf083)
Supplement: Supplementary_materials_fdaf083 [file supplementary_materials_fdaf083.docx]

# Supplementary Table 1. Summary of campaign materials as part of the ‘Find Cancer Early’ campaign in 2018, 2019, and 2020

|  | **2018 campaign** | **2019 campaign** | **2020 campaign^a^** |
| --- | --- | --- | --- |
| **Overall campaign period aired** | 8 April 2018 to 15 July 2018 | 4 November 2018 to 18 May 2019 | 2 February 2020 to 5 April 2020  10 May 2020 to 30 June 2020 |
| **Rural Doctors video advertisement description** | General practitioners from regional WA communities talking about the symptoms of common cancers, the importance of early cancer diagnosis, and seeking help when an unusual change in the body has been noticed | | |
| **Symptoms mentioned in the Rural Doctors advertisement** | - Nagging cough - Blood in your pee - Blood in your poo - Unusual lump, or swelling in your body - Changes in a spot on your skin - Unusual weight loss | | |
| **Rural Doctors video advertisement period aired** | 8 April 2018 to 15 July 2018 | 24 March 2019 to 18 May 2019 | 3 February 2020 to 5 April 2020  11 May to 30 June 2020 |
| **Rural Doctors video advertisement platform** | Television stations and digital/online platforms | | |
| **Radio advertisements description** | Aboriginal and non-Aboriginal male and female talent talking about the symptoms | | |
| **Symptoms discussed in radio advertisements** | - Coughed up blood - Nagging cough - Blood in your pee - Blood in your poo - Runny poo | | |
| **Radio advertisements period aired** | 8 April 2018 to 30 June 2018 | 24 March 2019 to 27 April 2019 | 29 February 2020 to 5 April  10 May 2020 to 7 June 2020 |
| **Radio advertisements platform** | Regional free-to-air radio stations | | |
| **Yellow Checklist description** | Checklist of the ten most common symptoms of the five most common cancers in Western Australia and the tagline, *“If you have (referring to the symptoms listed above) and you are over 40… Tell your doctor. The earlier cancer is found, the greater the chance of successful treatment.”* | | |
| **Symptoms discussed in the Yellow Checklist** | - Coughing up blood - Persistent cough - Becoming more short of breath - Blood in your pee - Blood in your poo - Problems peeing - Runny poo - An unusual pain, lump, or swelling anywhere in your body - A new or changed spot on your skin - Unexplained weight loss | | |
| **Yellow Checklist period aired** | 8 April 2018 to 30 June 2018 | 4 November 2018 to 11 May 2019 | 2 Feb 2020 to 28 March 2020  17 May to 28 June 2020 |
| **Yellow Checklist platform** | Regional newspapers and posters distributed to regional locations by Cancer Council Regional Education Officers | | |
| **Evaluation data collection dates** | 10 July 2018 to 3 September 2018 | 20 May 2019 to 5 June 2019 | 1 July 2020 to 1 August 2020 |
| ^a^ During the 2020 campaign, there was a 5 week hiatus in airing the advertisements when the World Health Organization declared COVID-19 a pandemic on 11 March 2020. | | | |

# Supplementary Table 2. Socio-demographic factors, cancer diagnosis history, and perspectives on earlier detection of cancer symptoms by survey year

| **Variable** | **2018** | **2019** | **2020** |
| --- | --- | --- | --- |
|  | **n=1707** | **n=1051** | **n=1052** |
|  | **n (%)** | **n (%)** | **n (%)** |
| **Age group** | | | |
| 40-64 years | 1130 (66.2) | 700 (66.6) | 668 (63.5) |
| 65+ years | 577 (33.8) | 351 (33.4) | 384 (36.5) |
| **Gender** | | | |
| Male | 776 (45.5) | 507 (48.2) | 505 (48.0) |
| Female | 931 (54.5) | 544 (51.8) | 547 (52.0) |
| **Region** | | | |
| Wheatbelt | 241 (14.1) | 150 (14.3) | 151 (14.4) |
| Great Southern | 240 (14.1) | 150 (14.3) | 150 (14.3) |
| Goldfields-Esperance | 240 (14.1) | 151 (14.4) | 150 (14.3) |
| Mid West-Gascoyne | 242 (14.2) | 150 (14.3) | 150 (14.3) |
| South West | 255 (14.9) | 150 (14.3) | 151 (14.4) |
| Kimberley | 240 (14.1) | 150 (14.3) | 150 (14.3) |
| Pilbara | 249 (14.6) | 150 (14.3) | 150 (14.3) |
| **Previous cancer diagnosis** | | | |
| No | 1325 (77.6) | 825 (78.5) | 796 (75.7) |
| Yes | 382 (22.4) | 226 (21.5) | 256 (24.3) |
| **Type of cancer^a^** | | | |
| Prostate | 37 (9.8) | 23 (10.3) | 32 (12.6) |
| Breast | 48 (12.7) | 37 (16.6) | 41 (16.2) |
| Skin | 169 (44.7) | 102 (45.7) | 120 (47.4) |
| Bowel | 32 (8.5) | 15 (6.7) | 16 (6.3) |
| Lung | 9 (2.4) | 5 (2.2) | 4 (1.6) |
| Other | 94 (24.9) | 49 (22.0) | 50 (19.8) |
| **Detecting cancer at an early stage means it can be treated more successfully** | | | |
| Agree | 1650 (98.4) | 1012 (98.2) | 1015 (98.1) |
| Disagree | 27 (1.6) | 18 (1.8) | 20 (1.9) |
| **Campaigns that raise awareness of cancer symptoms lead to earlier detection of cancer** | | | |
| Agree | 1185 (96.8) | 750 (96.9) | 766 (97.1) |
| Disagree | 39 (3.2) | 24 (3.1) | 23 (2.9) |
| **More should be done in your region to raise awareness of the symptoms of cancer** | | | |
| Agree | 811 (66.8) | 521 (69.7) | 560 (72.5) |
| Disagree | 403 (33.2) | 227 (30.3) | 212 (27.5) |
| **The state government should continue to fund cancer symptom awareness campaigns targeted towards regional people^b^** | | | |
| Agree | - | - | 727 (89.3) |
| Neural | - | - | 63 (7.7) |
| Disagree | - | - | 24 (3.0) |
| ^a^ Question restricted to participants who had reported having previously been diagnosed with cancer.  ^b^ Question only included in 2020 survey. | | | |

# Supplementary Table 3. Awareness levels of the ‘Rural Doctors’ advertisement and the ‘Yellow Checklist’, stratified by the pre-pandemic and pandemic waves

| **All waves (n=3810)** | | **‘Rural Doctors’ advertisement** | | | | **Total** |
| --- | --- | --- | --- | --- | --- | --- |
|  |  | **Unprompted**  **Awareness** | **Prompted**  **Awareness** | **Aware** | **Not Aware** |  |
| **‘Yellow Checklist’** | **Aware** | 156 (4.1) | 217 (5.7) | 373 (9.8) | 84 (2.2) | 457 (12.0) |
|  | **Not Aware** | 867 (22.8) | 1489 (39.1) | 2356 (61.8) | 997 (26.2) | 3353 (88.0) |
| **Total** | | 1023 (26.9) | 1706 (44.8) | 2729 (71.6) | 1081 (28.4) |  |
| **Pre-pandemic waves (n=2758)** | | **‘Rural Doctors’ advertisement** | | | | **Total** |
|  |  | **Unprompted**  **Awareness** | **Prompted**  **Awareness** | **Aware** | **Not Aware** |  |
| **‘Yellow Checklist’** | **Aware** | 111 (4.0) | 157 (5.7) | 268 (9.7) | 63 (2.3) | 331 (12.0) |
|  | **Not Aware** | 608 (22.0) | 1079 (39.1) | 1687 (61.2) | 740 (26.8) | 2427 (88.0) |
| **Total** | | 719 (26.1) | 1236 (44.8) | 1955 (70.9) | 803 (29.1) |  |
| **Pandemic wave (n=1052)** | | **‘Rural Doctors’ advertisement** | | | | **Total** |
|  |  | **Unprompted**  **Awareness** | **Prompted**  **Awareness** | **Aware** | **Not Aware** |  |
| **‘Yellow Checklist’** | **Aware** | 45 (4.3) | 60 (5.7) | 105 (10.0) | 21 (2.0) | 126 (12.0) |
|  | **Not Aware** | 259 (24.6) | 410 (39.0) | 669 (63.6) | 257 (24.4) | 926 (88.0) |
| **Total** | | 304 (28.9) | 470 (44.7) | 774 (73.6) | 278 (26.4) |  |

# Supplementary Table 4. Awareness of the ‘Find Cancer Early’ campaign by survey year

|  | **2018** | **2019** | **2020** |
| --- | --- | --- | --- |
|  | **n=1707** | **n=1051** | **n=1052** |
|  | **n (%)** | **n (%)** | **n (%)** |
| **Seen or heard an advertisement about cancer^a^** | 1306 (76.5) | 654 (62.2) | 787 (74.8) |
| **Awareness of campaign material^b^** | 1243 (72.8) | 775 (73.7) | 795 (75.6) |
| **No awareness of campaign material^b^** | 464 (27.2) | 276 (26.3) | 257 (24.4) |
| **‘Rural Doctors’ advertisement** |  |  |  |
| Unprompted awareness | 446 (26.1) | 273 (26.0) | 304 (28.9) |
| Prompted awareness | 749 (43.9) | 487 (46.3) | 470 (44.7) |
| Awareness | 1195 (70.0) | 760 (72.3) | 774 (73.6) |
| **‘Yellow Checklist’** |  |  |  |
| Awareness | 209 (12.2) | 122 (11.6) | 126 (12.0) |
| **Opinions about the ‘Find Cancer Early’ campaign^c^** |  |  |  |
| Easy to understand | 1213 (99.5) | 777 (99.2) | 767 (99.0) |
| Taught something new | 518 (43.0) | 313 (40.3) | 337 (43.5) |
| Uncomfortable (to view/watch) | 107 (8.7) | 67 (8.6) | 71 (9.1) |
| Believable | 1214 (99.2) | 769 (98.1) | 765 (98.5) |
| Relevant | 1017 (83.6) | 651 (83.0) | 634 (81.8) |
| Offensive | 26 (2.1) | 17 (2.2) | 20 (2.6) |
| ^a^ Advertisement not necessarily specific to the ‘Find Cancer Early’ campaign.  ^b^ Either the ‘Rural Doctors’ advertisement or the ‘Yellow Checklist’  ^c^ Questions restricted to participants who had awareness of the ‘Find Cancer Early’ campaign. | | | |

# Supplementary Table 5. Knowledge on the common symptoms of cancer by survey year

|  | **2018** | **2019** | **2020** |
| --- | --- | --- | --- |
|  | **n=1705** | **n=1051** | **n=1048** |
|  | **n (%) or M±SD** | **n (%) or M±SD** | **n (%) or M±SD** |
| **Number of cancer symptoms recalled** | 1.7±1.3 | 1.7±1.4 | 1.3±1.2 |
| **Cancer Symptom Knowledge** |  |  |  |
| Poorer (recalled <3 symptoms) | 1268 (74.4) | 780 (74.2) | 871 (83.1) |
| Better (recalled 3+ symptoms) | 437 (25.6) | 271 (25.8) | 177 (16.9) |
| **Cancer symptom recalled** |  |  |  |
| Coughing up blood^a^ | 63 (3.7) | 47 (4.5) | 35 (3.3) |
| Nagging cough | 124 (7.3) | 66 (6.3) | 42 (4.0) |
| Becoming more short of breath | 51 (3.0) | 38 (3.6) | 36 (3.4) |
| Blood in pee^a^ | 262 (15.4) | 153 (14.6) | 91 (8.7) |
| Blood in poo^a^ | 417 (24.5) | 229 (21.8) | 179 (17.1) |
| Problems peeing | 38 (2.2) | 23 (2.2) | 16 (1.5) |
| Runny/looser poo (diarrhoea) | 37 (2.2) | 30 (2.9) | 27 (2.6) |
| An unusual pain, lump, or swelling anywhere in your body | 926 (54.3) | 544 (51.8) | 487 (46.5) |
| Unexplained weight loss | 389 (22.8) | 259 (24.6) | 220 (21.0) |
| A new or changed spot on your skin | 657 (38.5) | 395 (37.6) | 254 (24.2) |
| Abbreviations: M: mean; SD: standard deviation | | | |
